# Effect of Electroconvulsive Therapy (ECT) on IL-1β, IP-10, IL-17, TNFα, IL-10 and Soluble IL-2 Receptor in Treatment-Resistant Schizophrenia (TRS) Patients—A Preliminary Study

**DOI:** 10.3390/jcm14093170

**Published:** 2025-05-03

**Authors:** Anna Maria Szota, Małgorzata Ćwiklińska-Jurkowska, Izabela Radajewska, Kinga Lis, Przemysław Grudzka, Wiktor Dróżdż

**Affiliations:** 1 Department of Psychiatry, Ludwig Rydygier Collegium Medicum in Bydgoszcz, Nicolaus Copernicus University in Torun, Curie-Skłodowskiej Street 9, 85-094 Bydgoszcz, Poland; przemyslaw.grudzka@cm.umk.pl (P.G.); wikdr@cm.umk.pl (W.D.); 2 Department of Biostatistics and Biomedical Systems Theory, Ludwig Rydygier Collegium Medicum in Bydgoszcz, Nicolaus Copernicus University in Torun, Jagiellonska Street 13-15, 85-067 Bydgoszcz, Poland; mjurkowska@cm.umk.pl; 3 Medical Center Gizińscy, Leśna Street 9, 85-676 Bydgoszcz, Poland; izabela.radajewska@gizinscy.pl; 4 Department of Alergology, Clinical Immunology and Internal Diseases, Ludwig Rydygier Collegium Medicum in Bydgoszcz, Nicolaus Copernicus University in Torun, Ujejskiego Street 75, 85-168 Bydgoszcz, Poland; kinga.lis@cm.umk.pl

**Keywords:** electroconvulsive therapy (ECT), treatment-resistant schizophrenia (TRS), cytokines, interleukins, antipsychotics

## Abstract

**Background**: Resistance to antipsychotic treatment in patients suffering from schizophrenia is linked to immune system disequilibrium. One effective therapeutic option for treatment-resistant schizophrenia is electroconvulsive therapy (ECT); however, its impact on cytokines remains poorly understood. The aim of this study is to evaluate the impact of ECT on cytokines (IL-1β, IP-10, IL-17, TNFα, IL-10, and soluble receptor for IL-2 (sIL-2R)) in TRS patients. Additionally, correlations between cytokine concentrations and schizophrenia symptoms severity are explored. **Methods**: Cytokine and receptor concentrations were measured in eight TRS patients before and after ECT and in 13 healthy participants from control group. The Positive and Negative Syndrome Scale (PANSS) was used to evaluate the severity of the symptoms. **Results**: Before ECT, TRS patients exhibited significantly higher concentrations of IL-1ß, IL-10, IL-17, and IP-10 compared to the control group, whereas no significant differences were observed in sIL-2R and TNF-α. In the TRS patients, ECT induced a significant reduction in IL-10, IL-17 and IP-10 levels, while IL-1β, TNF-α, and sIL-2R remained unchanged compared to pre-ECT. ECT also led to clinical improvement in schizophrenia symptoms, as measured by PANSS. Furthermore, correlations between cytokine levels and PANSS results were found. **Conclusions**: The above results suggest that clinical improvement in TRS patients following ECT is associated with immune modulation, especially with the steadiness between pro- and anti-inflammatory systems. However, further research is required to elucidate these mechanisms in greater detail.

## 1. Introduction

Schizophrenia is a debilitating psychotic disorder characterized by disturbances in emotions, perception, thinking, cognition and behaviour, resulting in poor social functioning and a reduced quality of life [1]. The underlying etiology of this disorder is still not fully understood; however, contemporary evidence supports the hypothesis that, regardless of the clinical stage of schizophrenia (acute, chronic, or treatment-resistant), the immune system is activated in this illness. During the first episode of psychosis, increased concentrations of pro-inflammatory cytokines (IL-1β, IL-6, TNF-α) and elevations of TGFβ and IFN-γ are reported [2,3]. Moreover, the exacerbation of schizophrenia symptoms is associated with raised concentrations of IFN-γ, TNF-α, IL-12, and sIL2R [4]. Increased concentrations of some pro-inflammatory cytokines (IL-6, IL-8, IL-17, IFN-γ), chemokines (C-C motif chemokine ligand 11 (CCL11), macrophage inflammatory protein-1 alpha (MIP-1), MCP-1), together with receptors (soluble tumour necrosis factor receptors 1 (sTNF-R1) and soluble tumour necrosis factor receptors 2 (sTNF-R2)), have been found during treatment with antipsychotics and may be prognostic biomarkers of TRS. Concurrently, TRS development is associated with a decreased production of interferon-gamma-induced protein-10 (IP-10), TNF-α, IL-2 and IL-4 [5,6,7,8]. Additionally, increased concentrations of IL-1β, IL-6, IL-10, IL-17, and sIL-2, and a reduction in TNF-α are associated with more severe positive symptoms of schizophrenia [9,10,11,12,13,14]. Elevations of IL-1β, IL-6, IL-8, IL-4, and TNF-α may also exacerbate negative symptoms of schizophrenia [9,15], whereas IL-6, together with IL-13 and IL-17, may intensify general symptoms in chronic schizophrenia [15,16]. The role of the immune system in schizophrenia has been confirmed by multiple data: (a) a lack of balance among different cytokines [4,6,17,18]; (b) changes in cytokine concentrations upon treatment with antipsychotic medication [19,20]; (c) microglial activation confirmed in animal studies and post mortem brain studies [21,22]; and (d) increased production of cytokines by microglia, astrocytes, and neurons [23].

The role of immune changes in schizophrenia has been extensively investigated [24,25], leading to the macrophage-T-lymphocyte theory of schizophrenia [25]. According to this theory, both pro-inflammatory (IRS) and anti-inflammatory (CIRS) systems are activated [25]. Pro-inflammatory molecules are produced by M1 macrophages (IL-1β, IL-6, and tumour necrosis factor-alpha [TNF-α]), Th-1 cells (IL-2, IL-12, and interferon-gamma [IFN-γ]) and Th-17 cells (IL-17), whereas CIRS activation induces T regulatory (iTreg) cells (with a higher level of IL-10 and transforming growth factor (TGF-β1) and a Th-2 shift (with increased levels in IL-4 and IL-5), collectively exerting an anti-inflammatory effect [5,26,27]. The overproduction of pro-inflammatory cytokines may have a harmful impact on neurons, leading to oxidative damage, reduced neurogenesis, and disrupted neuronal signalling. These effects are considered indicators of neuroprogression in schizophrenia. Moreover, cytokines such as IL-6 and IL-1β, which exert inflammation, and TNF-α stimulate the liver to produce acute-phase proteins (e.g., haptoglobin [Hp], alpha-1 antitrypsin [α1-AT], and alpha-2 macroglobulin [α2M]). These proteins take part in the CIRS response as they have anti-inflammatory and anti-oxidative effects [28]. Furthermore, pro-inflammatory peripheral cytokines may access the brain, inducing a central inflammatory state and sustaining neuroinflammation. Cytokines activate microglia and astrocytes to produce IL-1α, IL-6, TNF, and IFN-γ, which may reduce the thickness and/or volume of the prefrontal cortex and hippocampus or induce neuronal and glial cell death [4,23,29]. Moreover, the increased production of IRS cytokines (e.g., IL-1 and IL-6) correlates with general psychopathological symptom severity [9], whereas the exaggeration of negative symptoms correlates with elevated TNF-α level [5,30]. The inflammatory IRS response is so exaggerated that the activation of the CIRS system, characterized by increased production of anti-inflammatory cytokines and neuroprotective defences, appears to be relatively inadequate in inhibiting IRS activation. Both the severity of schizophrenia symptoms and poor clinical outcomes are correlated with impairments in the CIRS response [31,32]. Even during remission of schizophrenia symptoms, the activity of both systems, the IRS and the CIRS, is sustained [18,26,33,34,35].

Current evidence indicates increased levels in several pro- and anti-inflammatory cytokines in TRS patients, including IL-2, IL-6, IL-10, IL-12, IL-17, beta2 microglobulin (B2M), IL-6 receptor (IL-6R), IL-1R antagonist (IL-1RA), interferon γ (IFN-γ), macrophage inflammatory protein 1α (MIP-1α), and chemokines (CCL2, CCL11) [5,6,26,36,37]. Nevertheless, no significant differences have been observed in the concentrations of brain-derived neurotrophic factor (BDNF), vascular endothelial growth factor (VEGF), TNF-α, IL-6, or TGF-β between TRS patients and control subjects [38,39], nor in the decreased levels in IL-5 in TRS patients [39].

Monotherapy with an antipsychotic remains the principal method of treatment for schizophrenia. First- and second-generation antipsychotics, used orally or parenterally as long-acting injections, are effective in reducing schizophrenia psychopathology but may cause many side effects (e.g., extrapyramidal symptoms; metabolic syndrome; sexual dysfunction; sedation; and cardiac, hematological, and gastrointestinal adverse effects). This may lead to non-adherence, which is relatively common among patients with schizophrenia and may result in worsening the course of the illness [40,41]. Another source of non-adherence among patients with schizophrenia could be a lack of insight, commonly associated with more pronounced symptomatology and poorer functional outcome [42,43].

Pharmacotherapy allows for remission in the majority of patients with schizophrenia [40]. Nonetheless, a meta-analysis of 12 studies including a total of 11,958 patients with first-episode schizophrenia revealed that almost a quarter developed treatment resistance in the early stages of treatment [44]. Estimated rates of TRS among patients with multiple episodes of the illness may be as high as 40–50% [45]. Therefore, strategies to improve the efficacy of schizophrenia therapy have been constantly investigated. Growing evidence indicates that the initiation of clozapine at an early stage in the presence of TRS is considered the gold standard [46]. Alternatives include combination therapy with other antipsychotics or augmentation with a mood stabilizer or an antidepressant [47]. ECT is internationally acknowledged as an effective and well-tolerated treatment for TRS [48,49]. Other neurostimulation methods, such as repetitive transcranial magnetic stimulation (rTMS) or transcranial direct current stimulation (tDCS), have less supporting evidence in TRS and have not been approved for widespread clinical use [50,51]. ECT has been found to be effective in the treatment of some neurological and neuropsychiatric conditions, including catatonia, behavioural symptoms of dementia, psychiatric symptoms accompanying multiple sclerosis, refractory status epilepticus, and Parkinson’s disease [52].

There is robust clinical evidence that ECT reduces the intensity of severe mental disorders, such as refractory schizophrenia [53], catatonic schizophrenia [54], and refractory major depressive disorder [55]. The effectiveness of ECT in patients with TRS treated concurrently with antipsychotics has been confirmed in numerous studies, including meta-analyses [53,56]. It may be suggested that the effectiveness of ECT in TRS patients, expressed as an improvement in schizophrenia symptoms, may be associated with a decrease in the concentration of pro-inflammatory cytokines, such as IL-6, IL-12, and TNF-α [38,39], along with increased anti-inflammatory markers, including IL-4, IL-10, and TGF-β1 [57]. Additionally, significant elevations of neurotrophic factors, such as BDNF and VEGF, induced by ECT have been positively correlated with the improvement of schizophrenia symptoms [38,58,59,60].

The above results and the perspective that TRS may be regarded as a neuroimmune disorder, make it reasonable to explore the associations between changes in serum cytokine levels [IL-1β, IP-10, IL-17, TNFα, IL-10, and soluble IL-2 receptor (sIL-2R)] and ECT in TRS patients. Additionally, we investigated correlations between the concentrations in these cytokines and the intensity of schizophrenia symptoms, as measured by the Positive and Negative Syndrome Scale (PANSS).

## 2. Materials and Methods

### 2.1. Subjects; Inclusion and Exclusion Criteria

The participants to the study group were recruited by psychiatrists from hospitalized patients, who were treated in the University Hospital No.1 in Bydgoszcz, Psychiatry Clinic, Poland. The subjects suffered from treatment-resistant schizophrenia and were referred for ECT by their doctors. Eight patients (five men and three women), aged 20–41 years (mean age 35) who had previously undergone at least two consecutive unsuccessful trials of antipsychotic treatment and satisfied criteria for treatment resistance, were included. The patients were diagnosed with either paranoid schizophrenia (six participants) or residual schizophrenia (two participants) and suffered from schizophrenia for between 3 and 23 years, (an average of 13.9 years). Five patients met criteria for ultra-treatment-resistant schizophrenia (UTRS) as they had not responded to clozapine [61,62]. That is why higher chlorpromazine equivalent antipsychotic doses had to be prescribed to them in comparison to patients treated with other antipsychotics. The mean antipsychotic dose was 1108 mg a day (chlorpromazine equivalent for the group) [63]. Very high combined doses of antipsychotics were used in four patients (chlorpromazine equivalent above 1000 mg/d); https://psychopharmacopeia.com/antipsychotic_conversion.php; accessed on 10 June 2024. Antipsychotic Dose Conversion Calculator. Five patients were diagnosed with past substance use disorder; two had nicotinism only. Three patients had a history of suicidal attempt/s and only one had been treated with ECT during a previous exacerbation. Thirteen healthy participants (two men and eleven women; mean age: 33 years) were in the control group. They had no history of past or current mental disorders.

From both groups (TRS patients and control), we excluded participants with serious neurological disease, current symptoms of addiction to alcohol or other psychoactive substance and those with autoimmune disease. Also, we excluded individuals who had any contraindications for conducting ECT (e.g., myocardial infarction within the last 3 months and severe heart problems; severe metabolic and lung disease or stroke within the last 4 weeks) or were not able to sign the informed consent for ECT.

Before any procedures were conducted in patients, all questions about the study were answered, and written informed consent was obtained from all the subjects participating in the study. The study was conducted in accordance with the Declaration of Helsinki. The Ethics Committee of Nicolaus Copernicus University in Torun Collegium Medicum in Bydgoszcz, Poland, also approved the protocol of the study (approval number KB 631/2018, obtained on 24 October 2018).

### 2.2. Electroconvulsive Therapy Procedures

ECT procedures were conducted in TRS patients after evaluation of their general health. Each patient had a physical and neurological examination, electrocardiogram (EEC), computed tomography (CT), and blood and urine tests. Also, PANSSs were conducted twice: before ECT and after the last session of ECT. A total of 3 sessions of ECT per week were conducted in each patient and the total number of sessions ranged from 11 to 15.

ECT was conducted under general anesthesia with i.v. thiopental (3–4 mg/kg, i.v.) and succinylcholine (0.5–1.5 mg/kg, i.v.), [64,65,66]. Thymatron System IV (Somatics; Lake Bluff, IL, USA) was used to generate waves. Stimulation electrodes were placed using a bilateral setup on the frontal–temporal region. One adequate seizure lasting more than 20 s with a high-amplitude and slow wave was induced for each session. Taking into consideration the clinical improvement and possible side effects caused by the procedure, the total number of ECT sessions for each patient was established.

### 2.3. Immunology Parameter Assay

Serum for assays of IL-1β, IL-17, IL-10, TNF-α, IP-10, and sIL-2R was sampled between 7.00 and 7.30 a.m. from all patients with schizophrenia and the control subjects. Blood collection was conducted twice, both pre-ECT and after the last ECT session. Blood was collected in a vacutainer with clot activator.

After 45 min, blood was centrifuged (3000 rpm for 10 min at 4 °C) and obtained serum was separated into sterile 1.5 mL microcentrifuge tubes and stored at −80 °C until assay. Blood was also collected, but only once (on the screening day) from healthy individuals. All samples were analyzed using ELISA kits [(IL-10, kit number: RD194572200R; IL-1β, kit number: RD194559200R; BioVendor—Laboratorni medicina a.s. Karase, Brno, Czech Republic); (IP-10, kit number: 850.950; IL-17, kit number: 850.940; sCD25 = sIL-2R, kit number: 950.500 Diaclone, Medics Biochemica Group, Besançon, France); TNF-α, kit number: SEA133Hu, Cloud-Clone Corp, Katy, TX, USA)] according to the manufacturer’s protocol. Concentrations of cytokines are measured in pg/mL. The same operator conducted all blood analyses.

### 2.4. Statistic Analysis

To support the limited number of patients, specific statistical analysis was performed. To improve the power of tests, the values were logarithmized to obtain normal distribution. Due to relatively small samples, Bayesian analysis was employed to increase the power [67]. For testing the differences between distributions of measures, pre and post Bayes factor dependent sample tests were applied. For testing the differences between distributions of measures pre-ECT and control group, or post-ECT and control group, Bayes factor independent sample tests were applied. Bayes factor inference on pairwise correlations was calculated to analyze dependencies between interleukins and PANSS scores. PS IMAGO PRO v. 9.0 package was used for calculations (https://en.predictivesolutions.pl/en/ps-imago-pro); accessed on 12 December 2024. 

## 3. Results

### 3.1. Cytokine Concentrations and PANSS Score Changes Post-ECT

As a result of logarithmic normality of the PANSS scores (i.e., PANSS positive symptoms, PANSS negative symptoms, PANSS global psychopathology and PANSS total score) before and after ECT and similarly for all interleukins pre- and post-ECT and control subjects was obtained. Thus, Bayesian t-tests were applied for testing the null hypotheses of equality of means. Comparison of PANSS parameters before ECT (pre-ECT) and following ECT (post-ECT) is given in Table 1). According to this analysis, we found significant changes for all PANSS subscales in schizophrenic patients (Table 1) with *p*-values below <0.01. All PANSS scores decreased after ECT and this reflects substantial improvement of psychopathology under ECT.

Assuming that the hypothesis of equal averages is false, the probability of detecting a given effect using t- test is over 68% for all significant comparisons (see power of the test in Table 1).

As a result of the comparison between pre-ECT and post-ECT interleukin concentrations, significant differences were observed for IL-10, IL-17, and IP-10. More specifically, all three interleukins decreased following ECT (Table 1).

Comparing concentrations in interleukins between TRS patients pre-ECT and control subjects, significant differences (with *p*-values smaller than 0.001 or 0.005) were observed for IL-1ß, IL-10, IP-10, and IL-17. Concentrations in all these interleukins were lower in control subjects than in TRS patients before ECT (Table 2). Assuming that the hypothesis of equal averages is false, the probability of detecting a given effect using a t-test is over 99% for all significant comparisons (see power of the test in Table 2).

Comparison analysis between post-ECT and control subjects showed significant differences (with *p*-values less than <0.01) for two interleukins, IL-1ß and IL-10, the levels of which were higher in TRS patients post-ECT in comparison to control subjects **(**Table 3). Assuming that the hypothesis of equal averages is false, the probability of detecting a given effect using a t-test is over 99% for all significant comparisons (see power of the test in Table 3).

### 3.2. Cytokines and PANSS Score Correlations During ECT Procedures

In the tables, particular correlations (r) with marked significant values are given, according to the calculated probability of >|r| under the presumption of the null hypothesis of zero correlation.

For six concentrations of cytokines pre-ECT, the correlations were calculated. The values are presented in the lower triangle of Table 4, where the highest absolute value of −0.55 is obtained for the dependence between sIL-2R and IL-17, and the second correlation, equal to −0.51, is obtained for the pair of IL-10 and IL-17. Corresponding pairs for measures following ECT in the upper triangle of Table 4 have similar correlations: −0.51 for the pair sIL-2R with IL-17 and −0.52 for the correlation between IL-10 with IL-17. Association between interleukins following ECT is the highest for TNF-α and IL-17 (0.58, upper triangle of Table 4), the second one in turn is for the association between TNF-α and IL-10 (−0.54, upper triangle of Table 4). Only correlations between the same interleukins pre-ECT and post-ECT are significant at 0.05 or smaller for paired measures IL-1ß, IL-17, and sIL-2R (0.80, 0.76, and 0.75, respectively; diagonal of Table 4). The correlation between TNF-α pre-ECT and TNF-α post-ECT is the highest (0.84, diagonal of Table 4) and significant at the level smaller than <0.001.

Correlations between interleukins in the control subjects were calculated to obtain a comparative profile in healthy persons. Significant positive correlations were observed for the following pairs: IP-10 with IL-10 and IP-10 with sIL-2R (0,83 and 0,61, respectively; Table 5), while a significant negative correlation was detected for IL-1β and IL-17 pair (−0.57; Table 5). In the control group, higher associations between different interleukins were observed (at a significance level lower than <0.05) than for schizophrenic patients (both before and following ECT) (Table 5). The sign of correlation between IL-10 and IL-17 for controls was positive at 0.51 (Table 5), while corresponding correlations in TRS patients both before and following ECT were negative (−0.51 and −0.52, respectively; see lower and upper triangle of Table 4).

The highest correlation between interleukins and the PANSS scores for pre-ECT values was obtained for IL-1ß and PANSS positive symptoms (r = 0.64, *p* < 0.1 in Table 6). Additionally, IL-1ß was positively correlated with PANSS negative symptoms and PANSS total score (0.57 and 0.61, respectively).

The highest absolute value of correlation for post-ECT measures was detected between TNF-α and the PANSS negative symptoms, and this correlation was negative (−0.47 in Table 7). Similarly, the correlation for sIL-2R with PANSS total score was −0.47 (Table 7).

Correlations between changes from pre-ECT to post-ECT for six cytokines and four PANSS scores are presented in Table 8. The correlation between general psychopathology and changes in IL-10 is the highest and is positive (r = 0.71, *p* < 0.05), meaning a decrease in psychiatric scale is significantly connected to a decrease in IL-10. Additionally, a decrease in PANSS negative symptoms is connected to an IP-10 decrease (r = 0.7; *p* = 0.5) and TNF-α decrease (r = 0.68, *p* = 0.6).

## 4. Discussion

Resistance to treatment with antipsychotics in patients suffering from schizophrenia remains a clinical challenge. Data indicate that this impediment may be driven, among other factors, by the dysregulation of the immune system and may be ameliorated with ECT [68]. Since both the immunological background of drug resistance in patients with schizophrenia and the effects of ECT on cytokines have been insufficiently studied [38,39,57,58,59,66,68,69,70,71,72,72], we decided to examine immunology molecules such as IL-1β, IP-10, IL-17, IL-10, TNF-α, and sIL-2 receptor in TRS patients treated with ECT.

We observed increased concentrations in IL-1β, IL-10, IL-17, and IP-10 in TRS patients prior to ECT in comparison to the control group, whereas blood levels of sIL-2R and TNF-α remained not changed. Our findings on IL-1β and IL-17 are in line with previous results in TRS patients [6,18]. The increased concentration of IL-10 in our patients confirms data from Leboyer et al., 2015 [6], and Al.-Dujaili et al., 2020 [27], but contradicts Chen et al.’s 2023 [18] findings of decreased IL-10 in TRS patients. In the case of TNF-α, we found no significant group differences, which is consistent with Leboyer et al.’s 2021 [6] study, but contrasts with the decreased concentration of this cytokine reported by Chen et al., 2023, and Li et al., 2024 [18,73]. Additionally, we found no changes in sIL-2R concentration, which aligns with Chen et al.’s 2023 [18] results. Changes in IP-10 have not been previously evaluated in TRS patients. An interpretation of the discrepancies in the results obtained, due to limited data, the inhomogeneous population of TRS patients, and insufficient knowledge of drug resistance, is flimsy.

Earlier studies on both IL-1β and IL-17 suggested that these prominent cytokines may play a pivotal role in the etiology of schizophrenia, including TRS; however, this evidence is not conclusive. Some studies have revealed an elevated concentration of IL-1β in chronically ill patients who were stable, experiencing an acute relapse, recovering from exacerbation [4,16,17,74], or were drug-resistant [18]. In contrast, Potvin et al., 2008 [75], found no significant alterations in IL-1β concentration, and this was also reported in patients with chronic schizophrenia lasting more than six years [76]. With regard to IL-17, findings are even more inconsistent, as increased concentrations in IL-17 were observed in TRS patients [76], normal levels were found in chronic patients experiencing acute relapse [77] or those treated with clozapine [5], and a decreased concentration of IL-17 was found among chronic patients treated with different antipsychotics [78]. In this research, the concentrations of both IL-1β and IL-17 were higher in TRS patients than in healthy individuals. We may speculate that the increased production of IL-1β by M1 macrophages may have a dual effect on other cytokines. IL-1β in combination with IL-23 and IL-6 activates Th-17 cells, which produce the IL-17 responsible for inflammation. Activated Th-17 cells also produce IL-22 and TNF-α [6]. On the other hand, IL-1β may stimulate the liver to produce certain anti-inflammatory proteins (e.g., alpha-1 antitrypsin, haptoglobulin (Hp), alpha-2 macroglobulin), which may both inhibit production of IL-17 by Th-17 cells and stimulate the production of anti-inflammatory IL-10 by Treg cells [79]. In line with this, an increased concentration of IL-10 was found in our TRS patients. Moreover, IL-10 enhances the release of IL-1RA (antagonist of IL-1 receptor) from macrophages and inhibits Th-1, M1, and Th-17 cells [79]. As a result, IL-10 may inhibit the production of different cytokines, namely IL-1β by M1 cells, TNF-α by Th1 and Th-17 cells, and IL-17 by Th-17 cells, exerting an anti-inflammatory effect through these changes. The production of TNF-α in our patients is therefore inhibited, at least in part, by IL-10, as its concentration does not differ from the control group. We may hypothesize that in our study this anti-inflammatory effect of both IL-10 and liver proteins inadequately compensates for an exaggerated inflammatory response (IRS), and therefore increased concentrations in IL-1β and IL-17 in TRS patients are sustained.

Interpretation of the study results also requires consideration of the medication’s effect on the immune system. Our patients were treated with typical and atypical antipsychotics. Both risperidone and aripiprazole significantly decrease IL-1β and TNF-α [19,80] and significantly increase IL-10 in chronic schizophrenia patients [80,81]. With regard to clozapine, no significant differences have been observed in the concentrations in IL-10, IL-17 [5,82], or TNF-α [5,19] after treatment compared to before treatment in chronic schizophrenia patients. However, other studies have reported opposing results. Giridharan et al., 2020 [83], found that clozapine decreases IL-1β and IL-17 concentrations, whereas Yuan et al., 2022 [20], observed that clozapine upregulates IL-17 and TNF-α and downregulates IL-1β in patients with schizophrenia undergoing constant treatment. Therefore, we may hypothesize that increased IL-17 concentration in our TRS patients could have resulted from the effects of clozapine, whereas risperidone and/or aripiprazole may increase the production of IL-10.

It was suggested that IL-1β may be found as a state marker linked with the intensity of schizophrenia symptoms [4]. The increased concentration of IL-1β h was found in first-episode psychosis (FEP) and in acutely relapsed (AR) patients and was normalized due to antipsychotic treatment. In contrast, TNF-α and sIL-2R were suspected as trait markers, as their concentrations remained elevated in cases with exacerbation and following antipsychotic treatment [4]. In our TRS patients, IL-1β concentration remained elevated after antipsychotic treatment and did not decrease to values similar in the control group. However, concentrations in both TNF-α and sIL-2R were not different from the control group. Therefore, although the number of our patients was small and their heterogeneity substantial, we may suggest with caution, that IL-1β may not be a state marker, and both TNF-α and sIL-2R may not be trait markers in TRS patients.

In this study, we also investigated potential correlations between cytokines and PANSS scores before and after ECT. Significant positive correlations between IL-1β and PANSS positive symptoms (r = 0.64, *p* < 0.1), and with PANSS negative symptoms and PANSS total score (0.57 and 0.61, respectively), in TRS patients before ECT were found. This indicates that increased IL-1β in our patients was correlated with higher severity of schizophrenia symptoms, both positive and negative. Regarding other cytokines (IL-17, IL-10, TNF-α, IP-10 and sIL-2R), no correlations with any of the PANSSs were found. These results may be perceived as pioneering, as other cytokines, i.e., IL-2 [5,9,39], IL-12, IL-5, TGF-1β [39], and chemokine CCL11 [5] were within the area of the researchers’ interest.

Multiple correlations between cytokines and types of schizophrenia were noticed in first-episode and drug-naïve (FEDN) schizophrenia patients, and in patients with chronic schizophrenia. Increased concentrations of IL-1β, IL-6, IL-10, IL-17, sIL-2R, and IL-33, but also decreased concentrations of TNF-α, were similarly associated with more severe positive symptoms of schizophrenia [9,10,11,12,13,14]. More intensive negative symptoms are seen in chronic schizophrenia patients with higher concentrations of IL-1β, IL-6, TNF-α, IL-8, IL-4, IL-10, and sIL-2R, as well as in patients with decreased concentrations of IL-17 [9,15,84,85]. Furthermore, positive associations between increased concentrations of IL-6, sIL-2R, and IL-17, and decreased concentrations of TNF-α with general psychopathology PANSS sub-scores were reported [15,16]. Furthermore, a positive correlation between the total PANSS score and increased concentrations of IL-1β, sIL-2R, IL-13, IL-6, and IL-17 in chronic patients with schizophrenia was found [9,84,86]. Cytokine concentrations may be related to some behavioural problems and cognitive abilities in schizophrenia. For example, aggressive behaviour occurs more commonly in subjects with higher concentrations in IL-17 and IL-10 [14,84], whereas poorer cognitive abilities were associated with higher IL-10 [87] and lower TNF-α in chronic schizophrenia patients [13].

Analysis of the effect of ECT on cytokines showed a significant amelioration of IL-10 (*p* < 0.001), IL-17 (*p* = 0.026), and IP-10 (*p* = 0.038) in TRS patients post-ECT versus pre-ECT. With regard to IL-1β, TNF-α, and sIL-2R, the difference post-ECT compared to pre-ECT was not statistically significant. The concentration of IL-1β post-ECT did not decrease; however, it was higher compared to the control group (*p* < 0.01). This suggests that increased IL-1β in patients with schizophrenia may be responsible, not only for activation and maintaining an inflammatory state underlying the illness, but also for resistance to antipsychotics. An increased concentration of TNF-α was noted both in patients with chronic schizophrenia [17] and in patients chronically treated with clozapine, as this drug stimulates the production of TNF-α and IL-17 [20]. Our TRS patients had elevated levels of both IL-17 and IL-1β before ECT. We may hypothesize that clozapine and IL-1β activate Th-17 cells, which produce IL-17, IL-22, and TNF-α [6]. ECT reduced the concentration of IL-17, whereas IL-1β remained unchanged, as expected. Therefore, TNF-α and the increased concentration of IL-1β may cause the shading of TNF-α receptors in plasma. This could be the reason for the increase in sTNF-R1 and sTNF-R2 receptors [88]. Since both receptors for TNF-α act as decoy receptors, they attenuate TNF-α signalling, and thus, toxic concentrations of TNF-α do not occur [26]. The existence of such a relation between IL-1β and TNF-α and the fact that high levels of IL-1β were maintained even post-ECT suggest that the specific inflammatory pathway between IL-1β and TNF-α was not affected by ECT.

There are no available studies reporting the impact of ECT on cytokines studied by us in TRS patients. As for other cytokines, the increased concentration of TGF-β1 [57]; decreased concentrations in IL-6, IL-12, and IL-10 [39]; and a lack of change in IL-5 and TGF-β1 [39] after ECT in TRS patients were found.

Meta-analyses of the effect of antipsychotic treatment on cytokines (Table 9) show that both pro- and anti-inflammatory responses are influenced, and more components are affected in the FEP group than in the whole group of schizophrenic patients. This may mean that the immune reaction to antipsychotic treatment weakens during the course of the illness. On the other hand, data from a few studies point to the possibility that ECT, even in treatment-resistant patients, may exert a more extensive influence on pro- and anti-inflammatory cytokines, enabling the re-shaping of the immune response (Table 9) [38,39,57,89,90].

Aside from the changes in cytokines following ECT, we discovered a moderate negative correlation between TNF-α and PANSS negative symptoms (−0.47), and between sIL-2R and the PANSS total score (−0.47). This result displays that the increased concentration of TNF-α may be linked with the attenuation of negative symptoms of schizophrenia, whereas a decrease in the PANSS total score (clinical improvement in schizophrenia) seems to be combined with an increase in sIL-2R concentration. As the levels of IL-1β, TNF-α, and sIL-2R post-ECT versus before ECT were not statistically significant, and the concentration of IL-1β was higher than in control individuals, we may speculate that potential changes in the concentrations in these cytokines may result from the influence of other cytokines (pro- and anti-inflammatory) rather than from ECT itself. Moreover, this indicates that IL-1β may be involved in the maintenance of inflammation and drug resistance development. Earlier findings have revealed that a significant association exists between an increase in the PANSS score (exacerbation of schizophrenia symptoms) and higher concentrations of IL-17 and IL-10 [12]. Therefore, the reduction in IL-17 and IL-10, following ECT seen in this study, may have a long-term beneficial effect on schizophrenia symptoms. Moreover, this hypothesis may be strengthened by the fact that the IL23/IL-17 pathway may potentially become a therapeutic target for patients with TRS [6]. The interpretation of the clinical significance of IP-10 changes is currently not possible due to conflicting results from two available studies [91,92].

This study noted a statistically significant decrease (*p* < 0.001) in all the PANSS subscales in TRS patients, which points to a substantial reduction in schizophrenia symptoms following ECT. This was observed in previous studies with TRS patients who were treated with different antipsychotics (including clozapine, as in our patients) along with ECT. The improvement in schizophrenia symptoms, expressed as a reduction in the PANSS score, was between 40 and 71%, regardless of medication(s) dose(s), duration of illness in years, years of treatment, and the number of ECT sessions. [93,94,95,96]. The majority of our patients were on clozapine. Therefore, the significant improvement in schizophrenia symptomatology as a consequence of ECT treatment might be both expected and beneficial for the patients. The superior clinical efficacy of ECT in schizophrenia and TRS patients supports its incorporation into treatment standards and its wide availability in this indication. However, data indicate that this is not the case, and it varies significantly among countries and even among different regions [48]. Clozapine-resistant schizophrenia remains a challenging clinical situation as limited evidence from randomized trials of treatment options exists.

However, augmentation with ECT for positive symptoms and suicidality in individuals with schizophrenia follows expert consensus recommendations [47]. Our findings may point to the possibility that ECT exerts a more pronounced impact on the immune system and psychopathology than therapy with antipsychotics only in ultra-resistant schizophrenia. This may indicate that ECT should be incorporated through the organization of mental health systems, as addressed by Sampogna et al., 2024 [97].

ECT has been used in many neuropsychiatric disorders, including the treatment of catatonia. Current diagnostic classifications of mental disorders (i.e., DSM-5 and ICD-11) regard catatonia as a separate category and only a fraction of cases belong to schizophrenia [98]. Notably, no patient from our group met the criteria for catatonia. A systematic review of 31 publications on catatonia treatments revealed that the rates of patients with this illness who improved under ECT were similar to that of lorazepam administration, i.e., 60–100%. [99]. Other authors reported 83% to 90% response rates in catatonia with ECT [98]. Neuroleptic malignant syndrome (NMS) is conceptualized by many experts as a malignant form of catatonia. Interestingly, many laboratory abnormalities indicating the acute phase response (APR) in NMS have been reported: c-reactive protein (CRP), procalcitonin, erythrocyte sedimentation rate, D-dimer, fibrinogen, and alpha-1 chymotrypsin increase together with an elevation of interleukin-6. It is hypothesized that APR activation in NMS may influence central dopaminergic transmission [100].

Due to the low quality of studies on ECT in the treatment of behavioural symptoms of dementia there are no evidence-based recommendations in this field. However, ECT temporarily influenced depressive and psychotic symptoms. In patients with Lewy body dementia, the intensity of visual hallucinations, delusions and other psychiatric symptoms were induced by ECT. Also, a decrease in symptoms of mania, aggression, and agitation in patients with mild to severe dementia was observed. Nevertheless, symptoms of confusion and other forms of cognitive decline were present in some patients, but other subjects showed some improvement [101]. ECT could also ameliorate psychiatric symptoms accompanying multiple sclerosis [102]. A systematic review of evidence on ECT in refractory status epilepticus included 14 publications and retrospectively described 19 patients. The majority of them (almost 58%) improved and seizure control was maintained in a substantial fraction of cases for 2 weeks to 3 months. As a low grade of evidence has been reported, the routine use of ECT cannot be recommended [103]. According to experts, ECT is underutilized in Parkinson’s disease despite data on the positive effects of the procedure in patients with suboptimal effects of pharmacological treatment. Moreover, ECT is safe, although the duration of improvement varies among patients [104].

Cochrane meta-analysis of 15 studies on ECT in TRS, which included a total of 1285 patients, showed that ECT alone caused an improvement in terms of clinical response; however, no advantage or disadvantage of ECT, in addition to standard care for other outcomes, such as psychopathology scores, was found [105]. It is worth noting, in the context of our study results, a prospective observation of 253 TRS patients on combination therapy with ECT and flupenthixol revealed that more than half of the subjects responded to this therapy, and both the long duration of the illness and absence of affective symptoms did not herald resistance to ECT [106].

As mentioned earlier, TRS development is associated with a disequilibrium between the inflammatory IRS system and the anti-inflammatory CIRS system activation. ECT stands as an effective and pivotal treatment in TRS patients, but the layout of the therapeutic outcome of ECT has been still investigated. Current data indicate that ECT, at least in part, may attenuate inflammation and activate an anti-inflammatory response [107]. In this study, we found that neither treatment with antipsychotic medication nor ECT decreased the concentration of IL-1β, which was significantly increased before treatment. Therefore, IL-1β may encourage the liver to produce acute-phase proteins that have an anti-inflammatory impact by stimulation of IL-10 and heme-oxygenase 1(HMOX1) production. Also, these proteins attenuate the release of the pro-inflammatory IL-17 [79]. This study found that ECT inhibited IL-17 production, as its concentration significantly decreased post-ECT compared to pre-ECT. An increase in TGF-β1 concentration may also contribute to a decrease in IL-17. ECT may also induce significant increase in TGF-β1, which is correlated with the amelioration of schizophrenia symptoms in TRS patients [57]. TGF-β1 may also inhibit the synthesis of other pro-inflammatory cytokines (IL-1β, TNF-α, IL-6, IL-12, and IL-13) and may stimulate the production of sIL-1RA, which combats inflammation. The inhibitory impact of TGF-β1 on TNF-α was not noted in the patients, and ECT did not change TNF-α concentration. Rojas et al., 2022 [108], proposed that ECT should reduce TNF-α concentration compared to pre-ECT in TRS patients [108]; however, an explanation for this discrepancy using the results of our study currently seems impossible.

Regarding IL-10, we noticed a significant decrease in the concentration of this cytokine post-ECT, which was positively correlated (r = 0.71, *p* < 0.05) with improvement of clinical state (a decrease in the PANSS general psychopathology sub-scale). We also found that increased IP-10 pre-ECT correlated with the inflammatory state in TRS patients. ECT caused a significant decline in IP-10 concentration. This change was positively connected with the attenuation of negative symptoms of schizophrenia (r = 0.7; *p* = 0.5). A possible explanation is that the increased production of IL-4 by Th2 cells induced by ECT inhibits production of IFN-γ, which in turn causes a decrease in IP-10 [57]. The impact of ECT on TRS patients described above indicates that the clinical improvement of schizophrenia symptoms measured by PANSSs is associated with a reduction in the concentrations of pro-inflammatory markers IL-17, IP-10, and anti-inflammatory IL-10. We reported a reduction in IL-6 and IL-12 induced by ECT with concomitant amelioration of schizophrenia symptoms [39]. The concentrations of cytokines may change with the number of ECT sessions conducted in patients with depression [109]. A significant increase in IL-6 was found after 1–2 sessions of ECT, followed by a decrease after the 5th session and further decrease to levels similar to baseline at the end of the ECT series. A comparable trend was noticed with regard to TNF-alpha [109]. This influence of ECT on cytokines indicates that multiple sessions of ECT may gradually reverse the immuno-inflammatory imbalance.

Taken together, the above mechanism underlying ECT’s effect on the immune system should be ascertained as hypothetical due to scarce data and requires longitudinal studies and further verification.

Considerations regarding the effectiveness of ECT should also include the impact of ECT on multiple systems. In brief, ECT generates intraneuronal and synaptic remodelling. As a result, some biochemical, neurophysiological, and structural changes may be observed [108,110,111]. The exact molecular mechanism of ECT has not been studied in depth, and inconsistent findings have been reported. Small sample sizes and lack of homogeneity in methodology are the main flaws of these studies. However, it seems that the effectiveness of ECT may be associated with its impact on epigenetic mechanisms. ECT may change the expression of the genes associated with schizophrenia development through the induction of the methylation of DNA, modification of histones (acetylation process), or through changes in the expression of microRNA (miRNA). At least six miRNAs (mir-181a; mir-137; mir-223; mir-107; mir-181b; mir-125b) were highly expressed in patients with first episodes of schizophrenia [112]. Additionally, ECT may change neural plasticity through an increase in the expression of induced pluripotent stem (iPS) genes such as Oct4, Sox2, c-Myc, and Klf4, and through methylation of the BDNF gene [113]. Also, ECT increases the expression of the TCF7 gene, which suppresses the function of the gene TCF7L2, which is associated with schizophrenia. These genes may enter the nucleus and induce neurogenesis (one of the requested, positive effects of ECT) through the canonical Wnt/B-catenin signalling pathway [114]. Moreover, ECT may improve cognitive function through modulation of the pathways regulated by the EP300 gene. The expression of the EP300 gene is associated with cognitive deficits in schizophrenia [115].

Current data suggest that attenuation of symptoms in TRS patients is also linked with the neurotrophic system. Increased concentrations in both BDNF and VEGF induced by ECT in TRS patients were positively correlated with an improvement of clinical symptoms (significant reduction in the PANSS positive, negative, and total scales) [38,58,69,71]. Also, increased BDNF levels may stimulate the synthesis of dopamine and its biochemical transformations, which are disrupted in TRS patients [71]. Additionally, BDNF and VEGF may modulate synaptic plasticity of hippocampus and have a beneficial effect on learning and memory [58]. Therefore, increased levels of neurotrophins induced by ECT may improve cognitive functions in TRS patients. Furthermore, ECT facilitate medications to enter the brain through the blood–brain barrier and express their therapeutic effects [116]. ECT may also cause changes in the grey matter volume [117] and microstructure of the limbic system, which correlate with the amelioration of schizophrenia symptoms [118,119]. Moreover, the functionality of the hormonal axis is restored as a beneficial impact of ECT.

## 5. Conclusions

In summary, our results confirm the presence of immune disequilibrium between IRS and CIRS systems in TRS patients and the potential role of IL-1β in development of drug-resistant schizophrenia. However, the concentration of IL-1β was increased in TRS patients even after antipsychotic treatment; therefore, this cytokine seems to not be a state marker, as suggested in previous studies. On the other hand, TNF-α and sIL-2R may not be trait markers in TRS patients since the concentrations in both were not different from the control group. Not surprisingly, we observed a meaningful clinical improvement in TRS patients following ECT, reflected in a significantly lower number of points in all the PANSS subscales and the PANSS total score. We also found that a TNF-α concentration increase post-ECT could be coupled with the attenuation of negative symptoms of schizophrenia, whereas a decrease in PANSS total score (clinical improvement in schizophrenia) may be linked with an increase in sIL-2R concentration.

## 6. Limitations

This study has some limitations. The main disadvantage is the small and heterogeneous group of TRS patients. Factors such as different diets, smoking of cigarettes, many stressful conditions, and lifestyle may have influenced the cytokine levels [120,121,122]. Moreover, the patients from this study were treated with antipsychotics and mood stabilizers before ECT, and this might have had an impact on immune balance (on both IRS and CIRS systems). However, the withdrawal of medications in TRS patients before ECT was not possible due to a lack of permission from the Ethics Committee. Furthermore, anesthesia and the injection of muscle relaxants before ECT may also influence cytokines’ levels [123]. Another concern is the frequency of the blood collection. In this study, blood was collected in fasted patients in the morning, one day before ECT and on the following day after finishing the ECT, but the number of ECT sessions in each patient was between 11 and 15. Last but not least, sex or menstrual cycle and existence of comorbid diseases may affect the concentration of immunology parameters, so they also should be controlled.

## 7. Future Perspectives

Future studies should involve two important designs: longitudinal observations with repeated cytokine measurements during the course of ECT to assess the dynamics of immune fluctuations and the measurement of immunology parameters in TRS patients treated with sham ECT versus real ECT.

## Figures and Tables

**Table 1:** Bayes factor dependent sample test pre-ECT vs. post-ECT for logarithmic measures.

Variable	N	Mean Difference	Std. Deviation	Std. Error Mean	Bayes Factor	t	df	Sig.(2-Tailed)	Power of the Test
IL-1β_pre IL-1β_post	8	0.15	0.23	0.08	1.16	1.77	7	0.120	0.989
IP-10_pre—IP-10_post	8	0.29	0.32	0.11	0.46	2.55	7	0.038	0.984
IL-17_pre—IL-17_post	8	0.17	0.17	0.06	0.33	2.81	7	0.026	0.681
IL-10_pre—IL-10_post	8	2.6	0.73	0.26	0	10.04	7	<0.001	1.000
sIL-2R_pre—sIL-2R_post	8	0.06	0.12	0.04	1.76	1.38	7	0.210	0.232
TNF-α_pre—TNF-α_post	8	0.01	0.11	0.04	3.74	0.31	7	0.765	0.082
PANSSPositive symptoms_pre—PANSSPositive symptoms_post	8	0.5	0,3	0.11	0.04	4.7	7	0.002	0.980
PANSS Negative symptoms_pre—PANSS Negative symptoms_post	8	0.38	0.21	0.08	0.03	5.06	7	0.001	0.991
PANSS Global psychopathology_pre -PANSS Global psychopathology_post	8	0.42	0.17	0.06	0.01	6.87	7	<0.001	1.000
PANSS Total score_pre—PANSS Total score	8	0.42	0.13	0.05	0	9.23	7	<0.001	1.000

Bayes factor: Null versus alternative hypothesis.

**Table 2:** Bayes factor independent sample test pre- ECT vs controls ^a^.

	Mean Difference	Pooled Std. Error Difference	Bayes Factor ^b^	t	df	Sig.(2-Tailed)	Power of the Test
	Control: Pre-ECT						
IL-1β	−1.49	0.32	0.007	−4.66	19	<0.001	1
IP-10	−45.86	7.74	0.001	−5.925	19	<0.001	0.999
IL-17	−51.37	16	0.102	−3.21	19	0.005	0.99
IL-10	−95.49	10.56	<0.001	−9.041	19	<0.001	0.99
sIL-2R	−74.66	397.07	3.18	−0.188	19	0.853	0.912
TNF-α	−0.38	0.56	2.666	−0.687	19	0.5	0.779

^a^ Rouder method: assumed unequal variance between groups. ^b^ Bayes factor: null versus alternative hypothesis.

**Table 3:** Bayes factor independent sample test following ECT versus controls ^a^.

	Mean Difference	Pooled Std. Error Difference	Bayes Factor ^b^	t	df	Sig.(2-Tailed)	Power of the Test
	Control: Post-ECT						
IL-1β	−1.06	0.327	0.095	−3.249	19	0.004	0.95
IP-10	−27.2	7.135	0.033	−3.812	19	0.001	0.944
IL-17	−25.83	16.352	1.228	−1.58	19	0.131	0.915
IL-10	−3.08	1.981	1.26	−1.557	19	0.136	0.907
sIL-2R	58.19	411.39	3.2	0.141	19	0.889	0.06
TNF-α	−0.25	0.496	2.906	−0.508	19	0.618	0.187

^a^ Rouder method: assumed unequal variance between groups. ^b^ Bayes factor: null versus alternative hypothesis.

**Table 4:** Bayes factor inference on pairwise correlations between cytokine concentrations. Pre-ECT results are represented in lower triangle and post-ECT values are marked with light grey background-upper triangle. Blue diagonal point variables measured before and after ECT.

Pre\Post	IL-1ß	IP-10	IL-17	IL-10	sIL-2R	TNF-α
IL-1ß	0.80 *	0.18	0.01	0.39	−0.02	0.15
IP-10	−0.09	0.43	0.10	−0.04	0.43	−0.07
IL-17	0.31	−0.06	0.76 *	−0.52	−0.51	0.58
IL-10	−0.02	0.33	−0.51	−0.08	−0.29	−0.54
sIL-2R	0.42	−0.09	−0.55	0.29	0.75 *	−0.15
TNF-α	0.19	−0.06	0.11	0.10	0.23	0.84 **

* or **—significance (0.05 or 0.001).

**Table 5:** Bayes factor inference on pairwise correlations between cytokine concentrations for control subjects.

Interleukin	IL-1β	IP-10	IL-17	IL-10	sIL-2R	TNF-α
IL-1β	1.00	−0.37	−0.57 *	−0.37	−0.12	−0.32
IP-10	−0.37	1.00	0.43	0.83 **	0.61 *	0.37
IL-17	−0.57 *	0.43	1.00	0.51	0.29	0.36
IL-10	−0.37	0.83 **	0.51	1.00	0.38	0.23
sIL-2R	−0.12	0.61 *	0.29	0.38	1.00	−0.22
TNF-α	−0.32	0.37	0.36	0.23	−0.22	1.00

* or **—significance (0.05 or 0.001).

**Table 6:** Bayes factor inference on pairwise correlations between PANSS scores and cytokine concentrations before ECT (n = 8).

Variable	PANSS Positive Symptoms	PANSS Negative Symptoms	PANSS General Psychopathology	PANSS Total Score
IL-1ß	0.64 *	0.57	0.49	0.61
IP-10	0.37	0.14	0.30	0.30
IL-17	0.03	0.02	−0.11	−0.04
IL-10	0.37	0.14	0.07	0.19
sIL-2R	0.44	0.06	0.42	0.37
TNF-α	0.35	0.39	0.62	0.53

*—significance (0.1).

**Table 7:** Results for correlations between cytokine concentrations with PANSS scores obtained after ECT (n = 8).

	PANSS Positive Symptoms	PANSS Negative Symptoms	PANSS General Psychopathology	PANSS Total Score
IL-1ß	−0.07	−0.30	−0.38	−0.39
IP-10	0.34	−0.12	−0.45	−0.24
IL-17	0.31	−0.04	0.05	0.12
IL-10	0.15	0.07	−0.05	0.07
sIL-2R	−0.39	−0.11	−0.40	−0.47
TNF-α	0.42	−0.47	0.31	0.20

**Table 8:** Bayes factor inference on correlations between changes in cytokines and psychiatric scales before and after ECT (n = 8).

Variable	PANSS Positive Symptoms_Diff	PANSS Negative Symptoms_Diff	PANSS General Psychopathology_Diff	PANSS Total Score_Diff
IL-1ß_Diff	0.30	0.07	−0.26	0.01
IP-10_Diff	−0.12	0.70 *	0.24	0.36
IL-17_Diff	0.22	0.27	−0.11	0.13
IL-10_Diff	0.33	0.16	0.71 **	0.56
sIL-2R_Diff	−0.14	0.08	−0.61	−0.37
TNF-α_Diff	0.41	0.68 *	0.25	0.60

* or **—significance levels, respectively (0.01 or 0.05).

**Table 9:** Changes in cytokines and their soluble receptors associated with antipsychotic treatment or ECT in individuals with schizophrenia and first episode psychosis.

Cytokine/Receptor	Function	Antipsychotics		ECT
		SCZ	FEP	
IL-1β/IL-1RA	Pro-inflammatory/ Anti-inflammatory	↓/0	↓/	0 ^/
IL-2/sIL-2R	Pro-inflammatory/ Anti-inflammatory	0/↑	0/	0 ^/
IL-6/sIL-6R	Pro-inflammatory	0/0	↓/	↓ #/
IL-12	Pro-inflammatory	↑		↓ #
IL-17	Pro-inflammatory		0	↓ ^
IP-10	Pro-inflammatory			↓ ^
IFN-γ	Pro-inflammatory	↓	↓	
TNF-α	Pro-inflammatory and Anti-inflammatory	0	↓	↓ **; 0 ^
IL-4	Adaptive and Anti-inflammatory	0	↓	↑ *
IL-5	Anti-inflammatory			0 #
IL-10	Anti-inflammatory	0	↓	↓ #
TGF-β1	Anti-inflammatory	0		↑ *; 0 #

SCZ—schizophrenia (data from Tourjman, [89]; FEP—First Episode Psychosis (data from Marcinowicz, [90]; ECT—electroconvulsive therapy (data from: Kartalci *; Valiuliene **; Szota #, [38,39,57], ^—this study); ↑ increase; ↓ decrease; 0—no effect; blank—no data.

## Data Availability

In justified cases, the corresponding author may send the source data included in this paper.

## Abbreviations

acutely relapsed (AR); alpha-1 antitrypsin (α1-AT); alpha-2 macroglobulin (α2M); beta2 microglobulin (B2M); blood–brain barrier (BBB); brain derived neurotrophic factor (BDNF); Brief Psychiatric Rating Scale (BPRS); central nervous system (CNS); Compensatory Immune Response System (CIRS); electroconvulsive therapy (ECT); first episode and drug-naïve schizophrenia (FEDN); first-episode psychosis (FEP), haptoglobin (Hp); heme-oxygenase 1(HMOX1); IL-6 receptor (IL-6R); IL-1R antagonist (IL-1RA); Inflammatory Response System (IRS); interferon γ (IFN-γ); macrophage inflammatory protein 1α (MIP-1α); myeloperoxidase (MPO); nuclear factor-kB (NF-kB); Positive and Negative Syndrome Scale (PANSS); schizophrenia (SZ); soluble IL-2 receptor (sIL-2R); transforming growth factor β1 (TGF-β1); treatment-resistant schizophrenia (TRS); tumour necrosis factor-alpha (TNF-α); vascular endothelial growth factor (VEGF).
